# Lemierre's syndrome secondary to community-acquired methicillin-resistant *Staphylococcus aureus* infection presenting with cardiac tamponade, a rare disease with a life-threatening presentation: a case report

**DOI:** 10.1186/s12245-014-0039-y

**Published:** 2014-09-26

**Authors:** Sonali Sihindi Chapa Gunatilake, Lakmini Gunarathna Yapa, Malinga Gallala, Rohitha Gamlath, Chaturaka Rodrigo, Harith Wimalaratna

**Affiliations:** 1 Department of Medicine, Teaching Hospital, Kandy 20000, Sri Lanka; 2 Department of Microbiology, Teaching Hospital, Kandy 20000, Sri Lanka; 3 Department of Clinical Medicine, Faculty of Medicine, University of Colombo, Colombo 00300, Sri Lanka

**Keywords:** Cardiac tamponade, Community acquired methicillin resistant Staphylococcus aureus, Fusobacterium necrophorum, Lemierre’s syndrome, Pericardiocentesis, Thrombophlebitis

## Abstract

**Background:**

Lemierre's syndrome is a rare condition characterized by thrombophlebitis of internal jugular vein, septicemia and septic metastatic infection of different organs. It is preceded by an oropharyngeal infection by anaerobic organisms. Community-acquired methicillin-resistant *Staphylococcus aureus* is now emerging as a causative organism in Lemierre's syndrome. Clinical manifestations vary depending on the organ system affected by the infection. Although rare, patients may present with life-threatening conditions such as cardiac tamponade.

**Case presentation:**

We report the first case, to our knowledge, of Lemierre's syndrome presenting with cardiac tamponade secondary to community-acquired methicillin-resistant *S. aureus* in a previously well 45-year-old Sri Lankan lady. Fever, sore throat and left-sided neck pain complicated with facial and left upper limb swelling were followed by severe shortness of breath for 24 h. There was tachycardia with pulsus paradoxus, low blood pressure and soft heart sounds. Pericardial effusion with cardiac tamponade was detected on echocardiogram and methicillin-resistant *S. aureus* species were isolated in both blood and pericardial fluid cultures. Venous duplex of neck veins and computed tomography scan of the neck showed thrombosis of left-sided internal jugular, external jugular, subclavian and axillary veins. Diagnosis of Lemierre's syndrome was made, and patient had a satisfactory recovery following emergency pericardiocentesis and a prolonged course of antibiotics.

**Conclusions:**

Although uncommon, Lemierre's syndrome is a life-threatening condition. Patients may present with cardiac tamponade secondary to purulent pericarditis in Lemierre's syndrome, where emergency pericardiocentesis is lifesaving. Community-acquired methicillin-resistant *S. aureus* is emerging as a causative agent in Lemierre's syndrome, and awareness is required amongst physicians for prompt diagnosis and appropriate empirical treatment to prevent mortality and morbidity associated with the disease.

## 1 Background

Lemierre's syndrome is a rare, life-threatening illness affecting healthy adolescents and young adults. It is characterized by thrombophlebitis of internal jugular vein (IJV) followed by septicemia and metastatic septic complications, secondary to an oropharyngeal infection [1]. The commonest causative organism is an anaerobic oral commensal *Fusobacterium necrophorum* (80% of the cases), but other oral commensal organisms are also being isolated in patients with Lemierre's syndrome [2]. Methicillin-resistant *Staphylococcus aureus* (MRSA), usually a nosocomial pathogen, has been reported increasingly amongst previously healthy individuals in the community causing serious skin and soft tissue infections [3]. Community-acquired MRSA infection causing Lemierre's syndrome is a rare but recognized association with much concern owing to the aggressiveness of the MRSA infection and limited treatment options [3]-[5].

Metastatic complications of Lemierre's syndrome are responsible for the mortality and morbidity of the affected individuals. Most commonly affected are the lungs. Although rare, it can manifest as mediastinitis or pericarditis [6],[7]. McLean and Tyler described the development of pericardial effusion and tamponade secondary to mediastinitis and anticoagulation in a 23-year-old post-partum female with Lemierre's syndrome [8] and Hoehn et al. reported a 10-month-old baby with Lemierre's syndrome who had developed hemorrhagic pericardial effusion and tamponade while receiving anticoagulation [9]. An extensive literature survey did not reveal Lemierre's syndrome first presenting with cardiac tamponade. We report the first case, to our knowledge, of Lemierre's syndrome presenting with life-threatening complication of cardiac tamponade, secondary to community-acquired methicillin-resistant *S. aureus* (CA-MRSA). Awareness of the disease, clinical presentations and causative organisms in Lemierre's syndrome amongst physicians would lead to timely intervention, administration of appropriate antibiotics and better patient outcome.

## 2 Case presentation

A 45-year-old previously well Sri Lankan lady was admitted to the Teaching Hospital, Kandy, Sri Lanka with severe shortness of breath and respiratory distress for a duration of 24 h.

She had experienced sore throat and mild fever 10 days back followed by left-sided neck pain for the last 5 days. Pain was not radiating but aggravated by neck movements. There was no associated trauma to the neck. Subsequently, the patient had noted progressive facial and left upper limb swelling without any color changes and motor or sensory disturbances. For the past 24 h, she had experienced progressive shortness of breath. There was no odynophagia, pleuritic chest pain, cough or hemoptysis. She denied constitutional symptoms, features suggestive of connective tissue disorder and past history of miscarriages or thromboembolic diseases. She had no recent travel history or sexual risk behaviors. Drug and allergic history were nil of note.

On examination, she was obese with a body mass index (BMI) of 28.4 kg/m^2^. Her axillary temperature was 100°F. Symmetrical facial swelling and left upper limb swelling were noted but had no color changes in the swollen areas. Ear and throat examinations were unremarkable. There was no cervical lymphadenopathy. Cord sign (an induration of the internal jugular vein beneath the anterior border of the sternocleidomastoid muscle) was positive. Her pulse rate was 130 beats per minute, low volume pulse noted with pulsus paradoxus, blood pressure was 80/50 mmHg, and had soft heart sounds. Respiratory rate was 48 cycles per minute; oxygen saturation was 93% on air and had bi-basal reduced air entry with stony dull percussion note. Abdominal and neurological system examinations were unremarkable.

Urgent investigations revealed sinus tachycardia with electrical alternans in the electrocardiogram and cardiomegaly with bilateral pleural effusions on chest roentgenogram. Echocardiogram showed evidence of pericardial tamponade with right atrial and right ventricular collapse. Pericardial fluid aspiration was performed under sterile conditions, removing 450 ml of straw color fluid. Subsequently, her respiratory distress settled and blood pressure normalized. Further investigations revealed the following: hemoglobin - 11 g/dL, white cell count - 17.7 × 10^3^/μL (neutrophils - 86.2%, lymphocytes - 6.9%, monocytes - 6.1%, eosinophils - 0.7%, basophils - 0.1%), and platelets - 486 × 10^3^/μL. Blood picture was suggestive of bacterial infection (polymophonuclear leucocytes with left shift up to band forms and mild thrombocytosis). Her erythrocyte sedimentation rate was 52 mm in the first hour (normal <20) and C-reactive protein level was 162 units (normal <5 units). Two blood cultures were positive for MRSA species. Pericardial fluid analysis showed the following: protein - 4 g/dL, white cells - 156/μL (neutrophils - 90%, lymphocytes - 9%), acid fast bacilli - not seen, liquid culture for mycobacterium tuberculosis - no growth, adenosine-deaminase level - 35 units/L, cytology - negative for malignant cells. Pericardial fluid culture revealed a growth of MRSA in 26 h of incubation.

**Figure 1:**
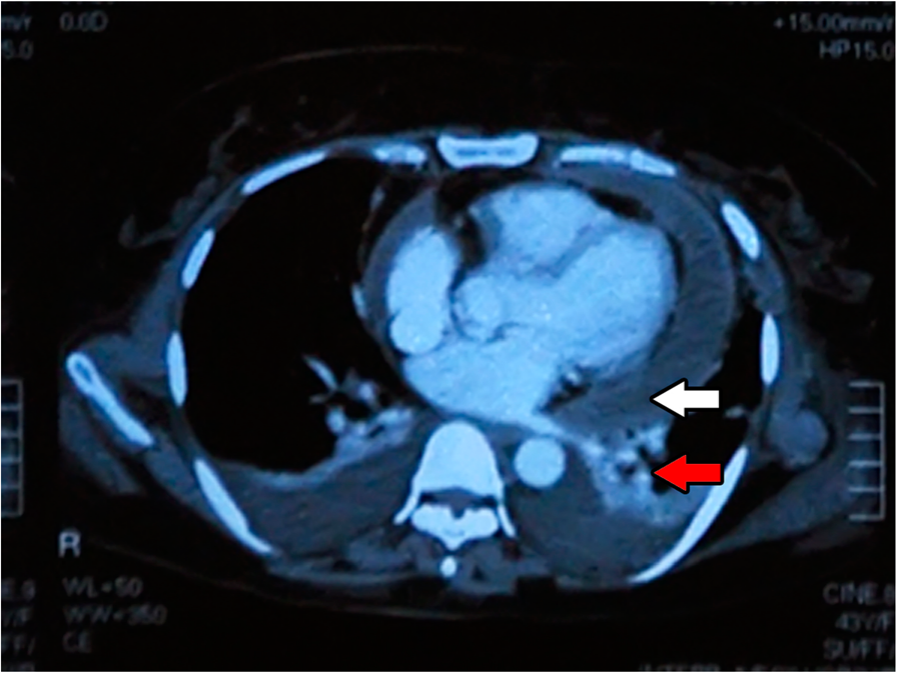
Contrast-enhanced CT of chest showing pericardial effusion (white arrow), bilateral pleural effusions and left-sided lung consolidation (red arrow).

Ultrasound scan and venous duplex scan of the neck showed thrombosis of left internal jugular, left external jugular, and left subclavian and left axillary veins with preserved flow in superior vena cava. Computed tomography (CT) scan of the neck and the chest showed similar findings to the venous duplex with additional moderate pericardial effusion (following first pericardiocentesis), bilateral pleural effusion, and left-sided consolidation of the lung (Figure 1). No lymphadenopathy or retropharyngeal abscess formation was noted in CT scan. Abdominal ultrasound scan was normal.

Her urine analysis, lactase dehydrogenase level and renal and liver function tests were normal. Nebulized samples for sputum acid fast bacilli staining and Mantoux test were negative. Serology for infectious mononucleosis, Epstein-barr virus, hepatitis B and C, and human immunodeficiency virus showed negative results. Thrombophilia screening was normal (anti-nuclear antibodies, anti-cardiolipin antibodies, homocysteine level, sickling test and factor V Leiden mutation).

The diagnosis of Lemierre's syndrome secondary to community-acquired MRSA was made with the evidence of history of sore throat, left internal jugular vein thrombosis, MRSA septicemia and distant metastatic infection involving pleura and pericardium, resulting in pleural and pericardial effusions. Pericardiocentesis was performed three times on day 0, day 1, and day 3 due to repeated tamponade effect. Patient was treated initially with intravenous meropenem and subsequently changed over to intravenous vancomycin (according to the antibiotic sensitivity of MRSA) and clindamycin. She showed dramatic response with complete resolution of symptoms and normal follow-up echocardiogram findings. Intravenous antibiotics were continued for 2 weeks and oral clindamycin was continued for further 4 weeks. Patient was initially given intravenous heparin followed by warfarin for a duration of 3 months. On further follow-up after 2 months, patient was completely asymptomatic without residual effects and repeat venous duplex of the neck veins showed recanalization and restoration of the blood flow in the previously thrombosed veins.

## 3 Discussion

Lemierre's syndrome, also known as post-anginal sepsis syndrome is a potentially life-threatening condition. It is characterized by initial oropharyngeal infection spreading to the pharyngeal spaces resulting in thrombophlebitis of the IJV. Infective organism spread from IJV causing septicemia and metastatic septic foci in the body.

The disease usually affects previously healthy adolescents or young adults aged 16 to 25 years old [2]. Prior to the advent of antimicrobial agents, Lemierre's syndrome carried a higher mortality and morbidity. But with the usage of antibiotics, the incidence has declined to one person per million per year and the disease was known to be a 'forgotten disease' [10]. Since 1990, many authors describe a resurgence of the disease, probably due to judicious use of antibiotics by primary care physicians, population changes, and increase in detection due to improved anaerobic blood culture techniques or increased awareness [10],[11].

Classical Lemierre's syndrome describes the oropharyngeal infection with the anaerobic gram-negative oral commensal *F. necrophorum* (in 80% of the cases) but many other organisms have also been isolated from diseased (*Bacteroids sp*., *Peptostreptococcus*, group B and C *Streptococcus*, *Enterococcus* and *Staphylococcus epidemidis*) [11],[12]. CA-MRSA has emerged as a causative agent resulting in Lemierre's syndrome since 2002, and the incidence is on the rise, probably owing to the increased prevalence of skin and soft tissue infections by CA-MRSA [12]-[14]. CA-MRSA is believed to be more virulent than nosocomial MRSA and is associated with more severe complications and thrombophilia. Thrombosis in transverse and superior sagittal sinuses, iliac veins, inferior vena cava, cavernous sinus and femoral and popliteal veins in association with CA-MRSA soft tissue infections are being recognized [3],[15]. Recent studies have identified similarities in pathogenic mechanisms amongst *F. necrophorum* and MRSA. Both organisms produce leucocidin, a protein which aid to lyse leucocytes and red cells to help immune evasion and bacterial replication [13],[14], which might explain the development of Lemierre's syndrome by both organisms.

Thrombophilic state induced by the infective organism causes thrombophlebitis of the IJV in classically described Lemierre's syndrome. It is clinically suspected when there is associated neck pain or cord sign (an induration of the internal jugular vein beneath the anterior border of the sternocleidomastoid muscle) or detected on imaging. IJV thrombosis is recognized in 30% to 70% of patients [16], and extension of the thrombus has been described in to the subclavian, axillary, brachiocephalic veins as well as in to the cerebral venous sinuses [3],[17]. Our patient also had thrombosis extending beyond internal jugular vein.

Metastatic infections can occur in any part of the body, namely bones, joints, meninges, liver, kidneys or spleen but pulmonary complications being the commonest (97%) [1],[2],[11]. It can be the presenting feature in Lemierre's syndrome. Pericardial involvement is reported rarely in the literature. Kachman and Vettese had described a 31-year-old male with Lemierre's syndrome presenting with right-sided pleural effusion and pericarditis [18], and Yuan et al. reported a case of a 26-year-old male with acute pericarditis as a rare manifestation of Lemierre's syndrome [6]. McLean and Tyler had published a case of a 23-year-old *primigravida* with Lemierre's syndrome who had developed cardiac tamponade secondary to mediastinitis and anticoagulation treatment. A 10-month-old child who had Lemierre's syndrome and hemorrhagic pericarditis while receiving anticoagulation was reported by Hoehn et al. [9], and Root et al. described another 10-month-old child with Lemierre's syndrome who developed purulent pericarditis and tamponade while on treatment [7]. Extensive literature survey did not reveal cardiac tamponade following purulent pericarditis as the first manifestation of Lemierre's syndrome up to date. Identification of this complication is important as there is high mortality of 30% to 75% associated with purulent pericarditis [19].

Combination of early diagnosis and timely administration of appropriate antibiotics are the key elements in treatment of Lemierre's syndrome. High-dose parenteral antibiotics according to the sensitivity of the organism should be administered over a prolonged period until the clot resolution takes place (at least 2 weeks intravenous followed by 4 to 6 weeks oral) considering the endovascular nature of the infection. Metronidazole, clindamycin, β-lactam/β-lactamase inhibitor and carbapenems have shown in vitro activity against *F. necrophorum*[11],[12]*.* For mixed infections, combination therapy is required. CA-MRSA is treated with vancomycin. Other options are daptomycin, linezolid and telavancin. Combination therapy in severe MRSA infection has not shown any benefit over monotherapy [12]. Drainage of the abscess or septic foci is needed where appropriate. Ligation or excision of the internal jugular vein is done only when there is evidence of continuous septic embolization despite adequate medical management [11]. Emergency situations should be handled as in the cited case above, where pericardiocentesis at the time of presentation was lifesaving.

Anticoagulation in Lemierre's syndrome is controversial as no randomized controlled trials exist. The clinician should weigh the risks and benefits of anticoagulation. Some indications for anticoagulation include thrombophilia, involvement of the cavernous sinus, occurrence of cerebral infarcts and refractory septic thrombophlebitis [12],[16]. In such patients, use of heparin followed by warfarin for 3 months had shown to reduce morbidity [1].

Lemierre's syndrome is a life-threatening condition but can be cured with timely appropriate intervention. During the pre-antibiotic era, the mortality was 90%, but with the usage of antibiotics, the mortality had dropped to 4% to 22% [11],[16]. Mortality and morbidity are mainly due to the septic metastatic complications.

The patient in this case report presented with life-threatening complication of cardiac tamponade following Lemierre's syndrome. She also had CA-MRSA cultured in blood as well as pericardial aspirate, which is not the classic organism described in the literature. High virulence of CA-MRSA would have been the reason for the severity of the disease and presentation with a life-threatening cardiac tamponade. High degree of suspicion from the onset to arrive at a diagnosis and timely, aggressive antibiotic treatment targeting MRSA saved her life without any morbidity.

## 4 Conclusions

Lemierre's syndrome remains a rare but an acute medical condition with considerable morbidity and potential mortality affecting young adolescents and adults. Patients may present with cardiac tamponade, an acute life-threatening complication of Lemierre's syndrome where emergency intervention is lifesaving. Community-acquired MRSA is emerging as a causative agent in Lemierre's syndrome in addition to the classically described anaerobic organisms. Awareness regarding the disease and causative organisms amongst physicians is needed to guide the empirical antibiotic treatment for a better outcome.

## 5 Consent

Informed written consent was obtained from the patient for publication of this case report and any accompanying images. A copy of the written consent is available for review by the Editor-in-chief of this journal.

## Abbreviations

CA-MRSA: community-acquired methicillin-resistant *Staphylococcus aureus*

CT: computed tomogram

IJV: internal jugular vein

MRSA: methicillin-resistant *Staphylococcus aureus*

## Competing interests

The authors declare that they have no competing interests.

## Authors' contributions

HW made the clinical diagnosis and clinical decisions in management and supervised the manuscript drafting. SSCG drafted the first manuscript and reviewed the literature. LGY made the microbiological diagnosis. HW, RG, SSCG, MG, and LGY were involved in direct management of the patient. CR assisted in manuscript drafting and revised it. All authors read and approved the final manuscript.

## Authors' information

HW (MBBS, MD, FRCP(Edin), FRCP(Lond), FCCP ) is a consultant physician. RG (MBBS, MD) is a senior registrar in medicine. SSCG (MBBS) is a registrar in medicine. MG (MBBS) is an intern medical officer in medicine. LGY (MBBS, MD) is a senior registrar in microbiology. All above authors are attached to the Teaching Hospital, Kandy, Sri Lanka. CR (MBBS, MD) is a lecturer in medicine at the Department of Clinical Medicine, Faculty of Medicine, University of Colombo.

## References

[B1] Kristensen LH Prag J Human necrobacillosis with special emphasis on Lemierre's syndrome Clin Infect Dis 2000 31 524 532 10.1086/313970 10987717

[B2] Baig M Rasheed J Subkowitz D Vieir J A review of Lemierre syndrome Internet J Infect Dis 2005 5 2 4457 http://ispub.com/IJID/5/2/4457 [http://ispub.com/IJID/5/2/4457]

[B3] Stauffer C Josiah AF Fortes M Menaker J Cole JW Lemierre syndrome secondary to community-acquired methicillin-resistant staphylococcus aureus infection associated with cavernous sinus thromboses J Emerg Med 2013 44 2 e177 e182 10.1016/j.jemermed.2012.02.075 22989693 PMC3527673

[B4] Zetola N Francis JS Nuermberger EL Bishai WR Community-acquired methicillin-resistant Staphylococcus aureus: an emerging threat Lancet Infect Dis 2005 5 275 286 10.1016/S1473-3099(05)70112-2 15854883

[B5] Lovy R, Ambler D, Fan W, Omron E: **Community-acquired methicillin-resistant*****Staphylococcus aureus*****infection presenting as Lemierre syndrome [abstract].** *Chest* 2006, **130**:295S.

[B6] http://content.onlinejacc.org/article.aspx?articleid=1856637 Yuan C, Choe D, Foster G: **Acute pericarditis as a rare manifestation of Lemierre's syndrome [abstract].** *J Am Coll Cardiol* 2014, **63**(12_s) []

[B7] Root RW Barrett TW Abramo TJ A 10-month-old with Lemierre syndrome complicated by purulent pericarditis Am J Emerg Med 2013 31 1 274 10.1016/j.ajem.2012.05.019 22809766

[B8] McLean AS Tyler K Cardiac tamponade in a postpartum woman with Lemierre's syndrome Anaesth Intensive Care 1998 26 5 582 583 9807617 10.1177/0310057X9802600518

[B9] Hoehn KS Capouya JD Daum RS Glikman D Gossett JG Hafzalah M Johnson D Marcinak J Lemierre-like syndrome caused by community-associated methicillin-resistant Staphylococcus aureus complicated by hemorrhagic pericarditis Pediatr Crit Care Med 2010 11 3 e32 e35 10.1097/PCC.0b013e3181b806cb 20453608

[B10] Riordan T Wilson M Lemierre's syndrome: more than historical curiosa Postgrad Med J 2004 80 328 334 10.1136/pgmj.2003.014274 15192164 PMC1743018

[B11] Eilbert W Singla N Lemierre's syndrome Int J Emerg Med 2013 6 40 10.1186/1865-1380-6-40 24152679 PMC4015694

[B12] Chanin JM Marcos LA Thompson BM Yesen RD Dunne WM Warren DK Santos CAQ Methicillin-resistant Staphylococcus aureus USA300 clone as a cause of Lemierre's syndrome J Clin Microbiol 2011 49 5 2063 2066 10.1128/JCM.02507-10 21430106 PMC3122675

[B13] Kadhiravan T Piramanayagam P Banga Gupta R Sharma SK Lemierre's syndrome due to community-acquired methicillin-resistant *Staphylococcus aureus* infection and presenting with orbital cellulitis: a case report J Med Case Reports 2008 2 374 10.1186/1752-1947-2-374 PMC2628934 19063718

[B14] Molloy A Towersey G Shackleton D Aali A Ash S The changing face of an old disease: case report of nonclassical Lemierre's syndrome caused by a Panton-Valentine leucocidin-positive methicillin-susceptible *Staphylococcus aureus* isolate J Clin Microbiol 2012 50 9 3144 10.1128/JCM.00939-12 22760040 PMC3421801

[B15] Kapur S Rutecki GW MRSA infections and thrombosis Consultant 2012 52 2 18

[B16] Wright WF Shiner CN Ribes JA Lemierre syndrome South Med J 2012 105 5 283 287 10.1097/SMJ.0b013e31825581ef 22561543

[B17] Min SK Park YH Cho YK Park JW Koh YH Seo TS Lemierre's syndrome: unusual cause of internal jugular vein thrombosis—a case report Angiology 2005 56 4 483 487 10.1177/000331970505600417 16079933

[B18] Kachman AM Vettese TE Lemierre syndrome: a common presentation of an uncommon disorder Hosp Physician 2001 37 10 52 54

[B19] Imazio M Cecchi E Demichelis B Salvatore I Demarie D Ghisio A Pomari F Coda L Belli R Trinchero R Indicators of poor prognosis of acute pericarditis Circulation 2007 115 2739 2744 10.1161/CIRCULATIONAHA.106.662114 17502574

